# Anti-pseudomonad Activity of Manuka Honey and Antibiotics in a Specialized *ex vivo* Model Simulating Cystic Fibrosis Lung Infection

**DOI:** 10.3389/fmicb.2019.00869

**Published:** 2019-04-24

**Authors:** Aled E. L. Roberts, Lydia C. Powell, Manon F. Pritchard, David W. Thomas, Rowena E. Jenkins

**Affiliations:** ^1^Department of Biomedical Sciences, Cardiff School of Health Sciences, Cardiff Metropolitan University, Cardiff, United Kingdom; ^2^Swansea University Medical School, Swansea University, Swansea, United Kingdom; ^3^School of Dentistry, College of Biomedical and Life Sciences, Cardiff University, Cardiff, United Kingdom

**Keywords:** *Pseudomonas aeruginosa*, biofilms, antimicrobial susceptibility testing, manuka honey, *ex vivo* model, cystic fibrosis

## Abstract

*Pseudomonas aeruginosa* causes problematic chronic lung infections in those suffering from cystic fibrosis. This is due to its antimicrobial resistance mechanisms and its ability to form robust biofilm communities with increased antimicrobial tolerances. Using novel antimicrobials or repurposing current ones is required in order to overcome these problems. Manuka honey is a natural antimicrobial agent that has been used for many decades in the treatment of chronic surface wounds with great success, particularly those infected with *P. aeruginosa*. Here we aim to determine whether the antimicrobial activity of manuka honey could potentially be repurposed to inhibit pulmonary *P. aeruginosa* infections using two *ex vivo* models. *P. aeruginosa* isolates (*n* = 28) from an international panel were tested for their susceptibility to manuka honey and clinically relevant antibiotics (ciprofloxacin, ceftazidime, and tobramycin), alone and in combination, using conventional antimicrobial susceptibility testing (AST). To increase clinical applicability, two *ex vivo* porcine lung (EVPL) models (using alveolar and bronchiolar tissue) were used to determine the anti-biofilm effects of manuka honey alone and in combination with antibiotics. All *P. aeruginosa* isolates were susceptible to manuka honey, however, varying incidences of resistance were seen against antibiotics. The combination of sub-inhibitory manuka honey and antibiotics using conventional AST had no effect on activity against the majority of isolates tested. Using the two *ex vivo* models, 64% (w/v) manuka honey inhibited many of the isolates where abnormally high concentrations of antibiotics could not. Typically, combinations of both manuka honey and antibiotics had increased antimicrobial activity. These results highlight the potential of manuka honey as a future antimicrobial for the treatment of pulmonary *P. aeruginosa* isolates, clearing potential infection reservoirs within the upper airway.

## Introduction

*Pseudomonas aeruginosa* is an opportunistic pathogen, capable of causing infections in various immunocompromised patient groups (de Bentzmann and Plésiat, 2011). Patients suffering with Cystic Fibrosis (CF) are at a heightened risk of *P. aeruginosa* infection (Lyczak et al., 2002). Amongst the many complications that affect the body of CF patients, mucus build-up within the lung results in chronic pulmonary colonization, with the nasal cavity and upper respiratory tract acting as reservoirs for recurrent infections (Siegel and Weiser, 2015). Up to 80% of CF patients will acquire a pulmonary *P. aeruginosa* infection, an event that is linked to poor patient prognosis and infection chronicity (Schelstraete et al., 2013). This link between *P. aeruginosa* and persistence, is due, in part, to the formation of biofilms by mucoid strains (Høiby et al., 2010).

Biofilm formation in *P. aeruginosa* leads to increased antimicrobial tolerance (AMT), an extra defensive strategy on top of the innate and acquired antimicrobial resistance (AMR) mechanisms it possesses (El Zowalaty et al., 2015). Eradication of *P. aeruginosa* from the CF lung is therefore not always possible, even when aggressive antimicrobial treatment regimens are used. There are few effective therapeutic options for pulmonary *P. aeruginosa* infections, with many patients harboring multi- drug resistant strains (López-Causapé et al., 2017). In order to provide respite care for these patients, novel antimicrobial agents with effective anti-biofilm effects must be identified (Worthington and Melander, 2013).

Recently, researchers have sought innovative antimicrobial strategies, utilizing natural products in order to combat AMR (Halstead et al., 2015; Harrison et al., 2015; Moloney, 2016). An antimicrobial agent which has demonstrable antimicrobial and anti-biofilm activity is manuka honey (Molan, 2016; White, 2016). It is well documented in wound microbiology that manuka honey is an effective treatment for chronic infections with exceptional activity against a range of AMR organisms (Mandal and Mandal, 2011; Maddocks and Jenkins, 2013). To date, two mechanisms of action have been identified: impairment of the cell division process described in *Staphylococcus aureus*, (Jenkins et al., 2011) and disruption of the cell envelope in *P. aeruginosa* (Roberts et al., 2012).

Previous manuka honey research has focussed on pathogens associated with wounds. However, many species found in wounds are synonymous with pulmonary infection. A recent study showed that manuka honey is capable of inhibiting the growth of multiple *P. aeruginosa* isolates (*n* = 56) of pulmonary origin (Jenkins et al., 2015), however, the methodologies used better represented acute infections as opposed to chronic biofilm-infections (European Committee for Antimicrobial Susceptibilty Testing, 2003). To ensure the best translational applicability for novel treatments, models that more accurately represent *in vivo* conditions are required (Roberts et al., 2015).

Recently, two *ex vivo* porcine lung (EVPL) models for the study of pulmonary *P. aeruginosa* biofilms have been described (Harrison et al., 2015; Harrison and Diggle, 2016). These provide a spatially structured *in vivo* environment with similar nutrient cues to those identified in CF sputum, allowing bacteria to form biofilms similar to those found in chronic lung infection (Palmer et al., 2005). This study is the first to utilize these more relevant models to determine the inhibitory efficacy of relevant antibiotics and medical grade manuka honey against a wide range of pulmonary *P. aeruginosa* isolates selected from an international reference panel (De Soyza et al., 2013). This focussed panel of isolates allows a wide range of pheno- and geno-types to be extensively tested, covering the wide diversity of *P. aeruginosa* isolates observed in the CF lung from commonly studied clones, to highly transmissible strains.

## Materials and Methods

### Ethics Statement

Materials required for the EVPL model were sourced from a local butcher as waste products of the food industry.

### Manuka Honey

Gamma-irradiated medical grade manuka honey (Medihoney; Integra Life Sciences) with a Unique Manuka Factor (UMF) rating of 12+ was used throughout.

### Bacterial Strains and Culture Conditions

Twenty eight *P. aeruginosa* Reference Panel (PaRP) isolates (De Soyza et al., 2013), were selected and covered a wide range of phenotypically and genotypically diverse isolates. Isolates were recovered from frozen stocks on Cation Adjusted (10 mg/L Mg^2+^ and 20 mg/L Ca^2+^) Mueller Hinton Agar (CA-MHA) for 24 h at 37°C. Log-phase and stationary phase cells were created by transferring single colonies to aliquots of Cation Adjusted Mueller Hinton Broth (CA-MHB) for 6 and 24 h, respectively, at 37°C.

### Minimum Inhibitory Concentrations (MICs) for Antimicrobial Agents

The Minimum Inhibitory Concentration (MIC) of manuka honey and clinically relevant antibiotics (Cystic Fibrosis Trust,  2009), was determined in accordance with microbroth dilution standards set out by the European Committee on Antimicrobial Susceptibility Testing (EUCAST; European Committee for Antimicrobial Susceptibilty Testing,  2003). Various doubling dilutions of manuka honey (1–32% w/v), ciprofloxacin (0.002–128 μg/ml), ceftazidime 0.03–2048 μg/ml), and tobramycin (0.016–1024 μg/ml) were used to determine the MICs. All results were derived from triplicate biological replicas.

### Determining Potential Interaction Between Antimicrobial Agents

To test for interactions between manuka honey and antibiotics, sub-inhibitory concentrations of honey (0.5, 0.25, and 0.125 of the MIC) were supplemented with various concentrations of antibiotics using a checkerboard methodology. Fractional Inhibitory Concentration Indices (FICI), which determine interplay between two antimicrobial agents were calculated. Mean FICI values from each of the sub-inhibitory manuka honey concentrations were used to allow a comprehensive dose-response analyses of total inhibition between the two antimicrobial agents and interpreted as: “synergistic” (FICI ≤ 0.5), “antagonistic” (FICI > 4.0), and “no interaction” (FICI > 0.5–4.0) (Odds, 2003). All results were derived from triplicate biological replicas for each of the sub-inhibitory manuka honey concentrations.

### Efficacy of Manuka Honey and Antibiotics Against PaRP Biofilms Grown in an *ex vivo* Porcine Lung (EVPL) Alveolar Model

The anti-biofilm effects of manuka honey and antibiotics were identified using an EVPL alveolar model (Harrison et al., 2014). Briefly, stationary growth phase bacterial cells were washed twice with Phosphate Buffered Saline (PBS) and re-suspended in an Artificial Sputum Media (ASM; Palmer et al., 2007) supplemented with 50 μg/ml ampicillin (ASM^Amp^) to a final cell density of ∼2 × 10^5^ cells/ml. Triplicate cubes (5 mm^3^) of EVPL alveolar tissue were dissected from the ventral surface of the left caudal lobe, washed three times with 1 ml Cell Culture Media supplemented with 50 μg/ml ampicillin (50% v/v RPMI 1640 and Dulbecco’s modified Eagle medium [DMEM]), and once with 1 ml ASM^Amp^. EVPL alveolar tissue cubes were placed on a bed of solidified ASM^Amp^ (using 0.8% agarose) in 24-well microtitre plates, submerged in 0.5 ml ASM^Amp^ and inoculated with 50 μl of cells using a 30-gauge needle. Cubes of EVPL alveolar tissue were incubated at 37°C. After 24 h, the cubes were rinsed with 1 ml PBS, removing loosely adhered cells, and treated with 0.5 ml of 0% (ASM^Amp^), 32%, or 64% w/v manuka honey supplemented with/without antibiotics (128 μg/ml ciprofloxacin, 2048 μg/ml ceftazidime, or 1024 μg/ml tobramycin) for 16 h. Increased concentrations of antimicrobial solutions were selected due to biofilms being inherently more tolerant of antimicrobial agents. Cubes of EVPL alveolar tissue were then rinsed with 1 ml PBS, and homogenized in 500 μl PBS with 7 mm × 2.8 mm metal beads using a Beadbug Microtube homogeniser (Benchmark Scientific) at 4000 rpm for 40 s. Total viable counts (TVCs) were completed for biofilm cells within in the homogenized samples. Biological replica data was obtained using triplicate independent porcine lungs obtained from the local butcher on separate occasions.

### Efficacy of Manuka Honey and Antibiotics Against Certain PaRP Biofilms Grown in an EVPL Bronchiole Model

In addition to the anti-biofilm effects studied using an EVPL alveolar model (simulating lower respiratory tract infections), the effect of manuka honey and antibiotics was studied against PaRP isolate #1 (LES B58, a representative transmissible strain), #17 (PAO1; a representative reference strain), and #28 (NH57388A; a mucoid phenotypically diverse strain) in an EVPL model using bronchiole tissue (simulating upper respiratory tract infections) (Harrison and Diggle, 2016). The transition from alveolar tissue to bronchiole tissue was to aid visualization of the tissue surface, as infection with *P. aeruginosa* isolates was highly destructive to alveolar tissue. Methodologies similar to the EVPL alveolar model were used, however, bronchiole sections were cleaned of alveolar tissue and dissected into triplicate 5 mm^2^ sections. These were washed, inoculated, and treated as above. TVCs of cells surviving treatment with each of the antimicrobial agents were determined; parallel samples for PaRP isolate #1 were prepared for Scanning Electron Microscopy (SEM) by fixing overnight in 2.5% [v/v] glutaraldehyde (4°C). Samples were rinsed in distilled water (x3) prior to critical-point drying, employing a gradual ascending series of ethanol dehydration steps. Finally, the samples underwent three changes in hexamethyldisilazane until the solution had evaporated. Samples were then sputter-coated with gold and imaged using the Tescan Vega SEM at 4 kV. SEM images were then processed in ImageJ software. Biological replica data was obtained using triplicate independent porcine lungs obtained from the local butcher on separate occasions.

### Statistical Analysis

Analysis of variance (ANOVA) was used to test the main effects of manuka honey and antibiotics on bacterial load with Dunnett’s *post hoc* test applied where applicable.

## Results

**FIGURE 1:**
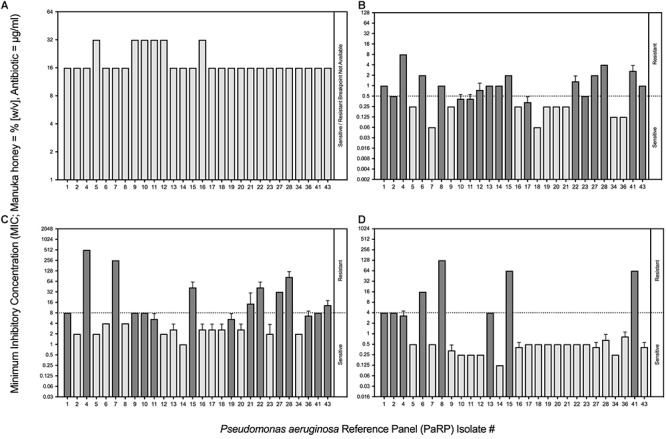
Minimum Inhibitory Concentrations (MICs) of *P. aeruginosa* Reference Panel (PaRP) isolates associated with Cystic Fibrosis (CF) to manuka honey (expressed as % [w/v]; **A**) and three clinically relevant antibiotics (expressed as μg/ml); ciprofloxacin **(B)**, ceftazidime **(C)** and tobramycin **(D)**. PaRP strains found to be sensitive/resistant to the antibiotics tested **(B–D)**, based on EUCAST breakpoints (dashed horizontal line), are colored light/dark gray, respectively (this does not apply to manuka honey **(A)** where there is no EUCAST breakpoint).

All PaRP isolates, using conventional antimicrobial susceptibility testing (AST), were inhibited by 16% or 32% (w/v) manuka honey (*n* = 22 and 6, respectively; Figure 1A). The majority of isolates with Minimum inhibitory concentration values of 32% w/v manuka honey (*n* = 5) were paired and/or sequential isolates taken at varying times from the same source (De Soyza et al., 2013). MIC values for ciprofloxacin, ceftazidime, and tobramycin varied above and below the EUCAST breakpoints (Figure 1B–D, respectively). The majority of isolates were sensitive to tobramycin (*n* = 20) and resistant to ciprofloxacin (*n* = 17) and ceftazidime (*n* = 15). PaRP isolate #1, 4, 15, and 41 were shown to be resistant to all tested antibiotics, however, they were inhibited with a lower manuka honey concentration [16% (w/v)].

**FIGURE 2:**
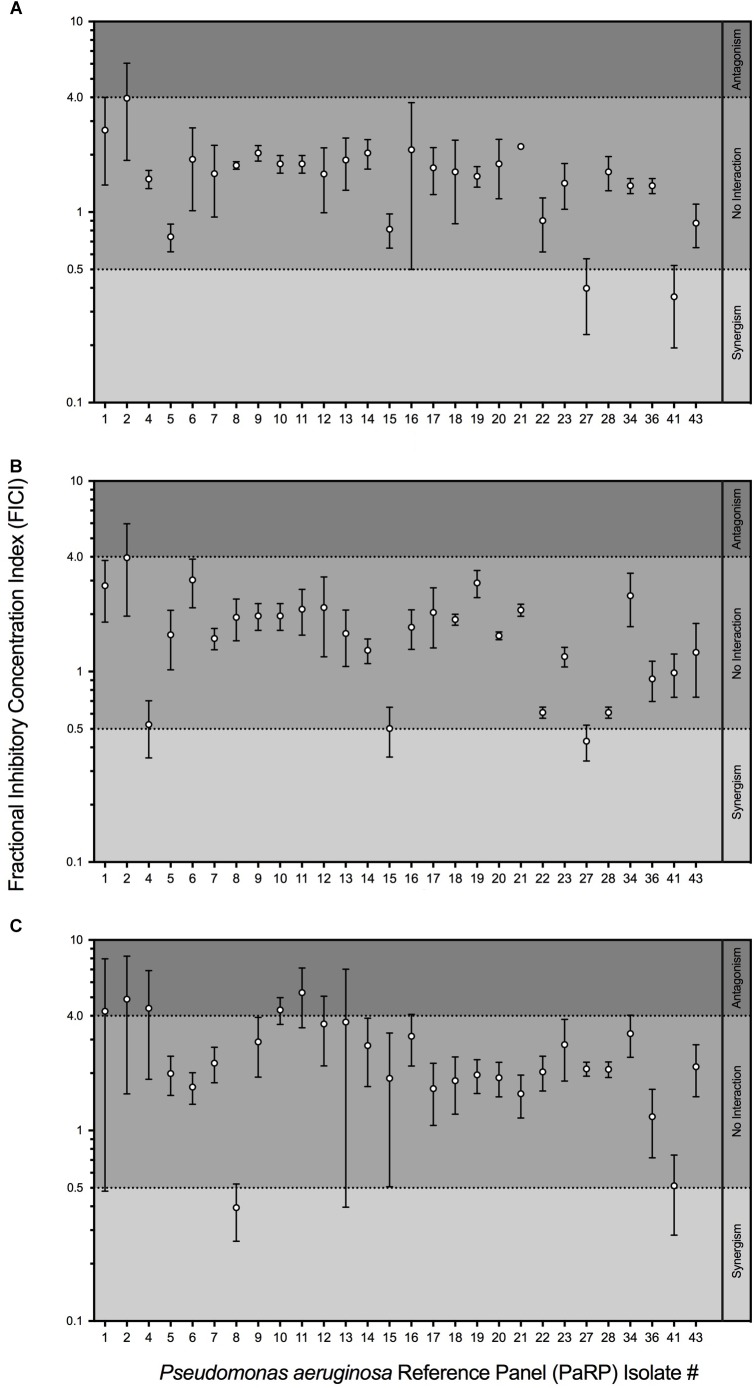
Fractional Inhibitory Concentration Index (FICI) of sub-inhibitory manuka honey concentrations supplemented with one of three clinically relevant antibiotics; ciprofloxacin **(A)**, ceftazidime **(B)** and tobramycin **(C)**. The interaction between manuka honey and antibiotics were interpreted as ’synergistic (FICI < 0.5), “antagonistic” (FICI > 4.0), and “no interaction” (FICI > 0.5–4.0).

Fractional inhibitory concentration indices values showed that manuka honey and ciprofloxacin had no interactive effects against the majority of PaRP isolates (*n* = 25; Figure 2A). Some isolate-specific interactions, dependent upon manuka honey concentration, were observed when FICI values crossed interaction “breakpoints” indicative of antagonism and synergism. As an example; various manuka honey and ciprofloxacin combinations worked synergistically against PaRP isolate #41 (Supplementary Figure 1) which was deemed resistant to ciprofloxacin (Figure 1B). FICI values for manuka honey and ceftazidime indicated no interaction between the two antimicrobial agents for the majority of isolates tested (*n* = 24; Figure 2B). Strain-specific interactions were again observed with some FICI values crossing interaction “breakpoints” indicating antagonism and synergism. As an example; 0.125 MIC manuka honey and ceftazidime worked synergistically against PaRP isolate #4 (Supplementary Figure 2) which was deemed resistant to ceftazidime (Figure 1C). Interactions between manuka honey and tobramycin were more varied, despite there being no discernible interaction against the majority of PaRP isolates tested (*n* = 19; Figure 2C). This was because FICI values for a larger proportion of isolates crossed antagonistic breakpoints due to two of the sub-inhibitory concentrations of manuka honey having antagonistic FICI values (Supplementary Figure 3). Synergism between tobramycin and 0.25 MIC manuka honey was observed against the tobramycin resistant isolate PaRP #41 (Supplementary Figure 3). Interestingly instances of synergy and antagonism, pushing the mean FICI value over the respective breakpoints were observed when low and high sub-inhibitory concentrations of manuka honey (0.125 and 0.5 MIC, respectively) were used (Supplementary Figure 3).

**FIGURE 3:**
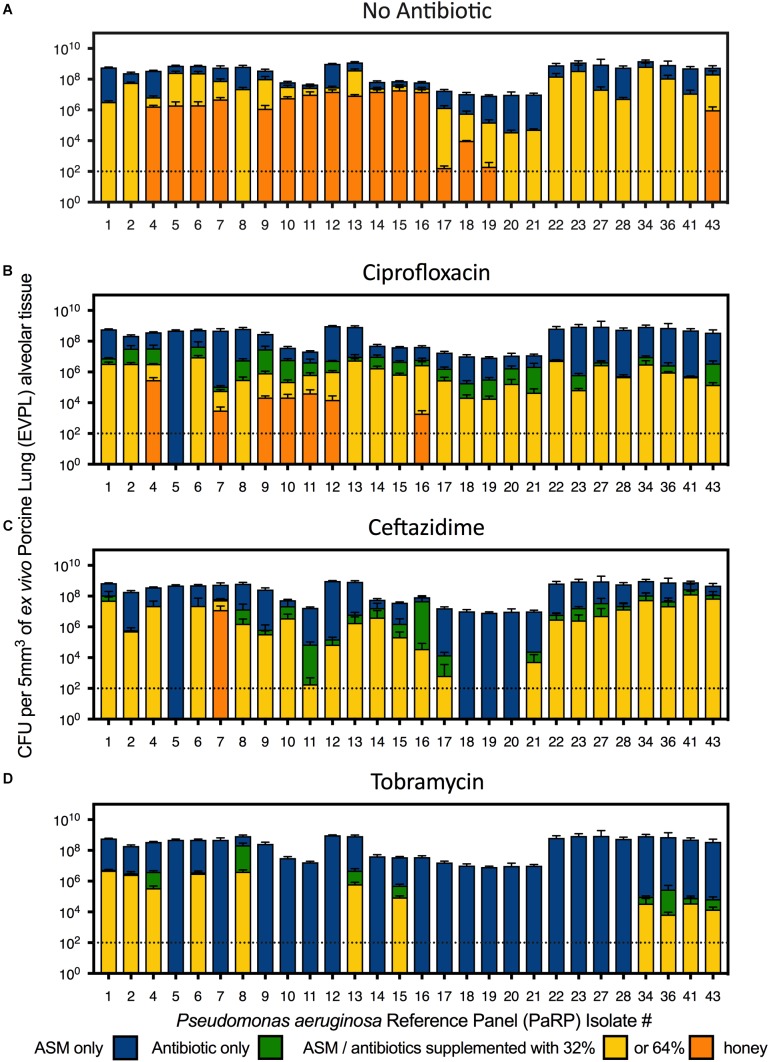
Viability of *P. aeruginosa* Reference Panel (PaRP) isolates grown in *ex vivo* Porcine Lung (EVPL) alveolar tissue for 24 h and treated with ASM **(A)**, ciprofloxacin **(B)**, ceftazidime **(C)**, and tobramycin **(D)** supplemented with 32 and 64% w/v manuka honey for 16 h. Dashed horizontal lines indicate the detection level of the EVPL model. Error bars denote Standard Deviation.

In the EVPL alveolar tissue, all PaRP isolates produced high cell density biofilms (>1 × 10^7^ cfu/5mm^3^; Figure 3A) with varying visible degrees of exopolysaccharide production (Supplementary Figure 4). Overall, manuka honey demonstrated significant inhibition of multiple PaRP isolates [*F*_(2,168)_ = 116.3, *p* < 0.0001], with a range of log-fold reductions being observed in the presence of 32% w/v manuka honey (strain dependent), in addition to the complete inhibition of multiple (*n* = 12) isolates with 64% w/v manuka honey (Figure 3A). This observation was not seen when]ciprofloxacin or ceftazidime were used alone (despite the high concentrations), however, tobramycin was effective at inhibiting the majority of isolates (*n* = 17; Figure 3D). When used in combination with ciprofloxacin, ceftazidime or tobramycin, manuka honey induced further statistically significant reductions in cellular viability [*F*_(2,168)_ = 27.68, *p* < 0.0001, *F*_(2,168)_ = 27.42, *p* < 0.0001, and *F*_(2,168)_ = 24.61, *p* < 0.0001, respectively], with an increased incidence rate of total inhibition increased (*n* = 21, 27, and 28, respectively). It was noted that against some PaRP strains, the addition of various antibiotics had a negative effect on 32% (w/v) manuka honey, slightly reducing its inhibitory efficacy, however, viability remained below that seen in control conditions with no treatment (Figure 3; e.g., ceftazidime and 32% w/v manuka honey had reduced efficacy against PaRP #1 compared to manuka honey alone, however, this activity was better than control).

**FIGURE 4:**
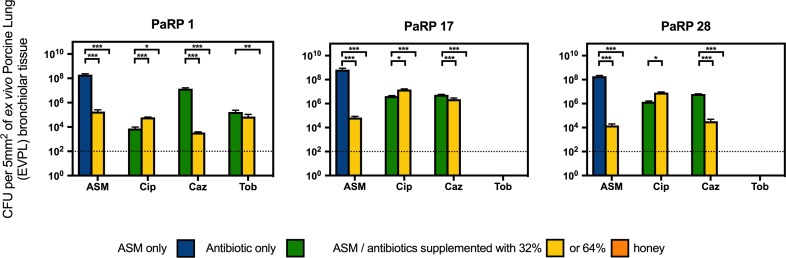
Viability of three *P. aeruginosa* Reference Panel (PaRP) isolates; #1, #17, and #28, grown in *ex vivo* Porcine Lung (EVPL) bronchiolar tissue for 24 h and treated with ASM or clinically relevant antibiotics (Cip; Ciprofloxacin, Tob; Tobramycin, and Caz; Ceftazidime) supplemented with 32% and 64% (w/v) manuka honey for 16 h. Dashed horizontal lines indicate the detection level of the model. Error bars denote Standard Deviation. Varying levels of statistical significance denoted by ^∗^, ^∗∗^, and ^∗∗∗^ (*P*≤0.05, 0.001, and 0.0001 respectively).

Three representative PaRP isolates grown on EVPL bronchiole tissue sections with ASM demonstrated dense biofilm growth (>1 × 10^8^ cfu/5 mm^2^; Figure 4). Manuka honey at 64% w/v completely inhibited cellular viability with and without antibiotics, with 3–4 log reductions observed in samples treated with 32% w/v manuka honey (Figure 4). Combinations of ciprofloxacin and manuka honey (32% w/v) caused a marginal (less than 1 log-fold difference), yet significant (*P* < 0.05) increase in cellular numbers compared to untreated samples (Figure 4). Conversely, ceftazidime and 32% (w/v) manuka honey caused significant reductions in viability (*P* < 0.001), however, the extent of inhibition observed was strain-dependent, with only marginal reductions against PaRP #17 (Figure 4). As tobramycin completely inhibited two of the three isolates tested, only one isolate could be studied. The viability of PaRP#1 was reduced when 64% manuka honey and tobramycin were combined (Figure 4).

**FIGURE 5:**
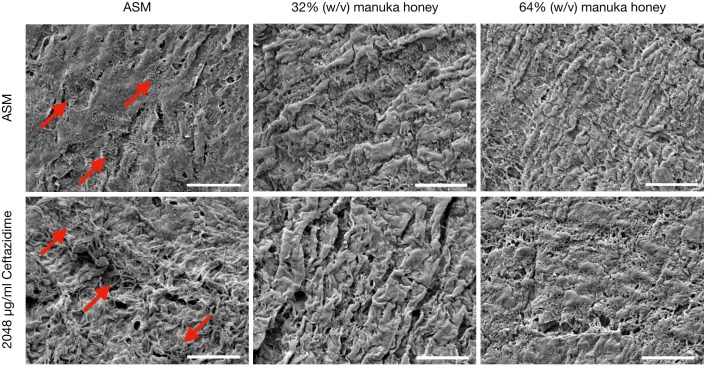
Scanning Electron Microscopy (SEM) images of *P. aeruginosa* Reference Panel (PaRP) isolate #11 (LES B58) recovered from *ex vivo* Porcine Lung (EVPL) bronchiole tissue samples grown in Artificial Sputum Media (ASM) with/without 2048 μg/ml ceftazidime and supplemented with/without 32/65% (w/v) manuka honey for 16 h. Images show bacteria cells (red arrows) and their subsequent inhibition with increasing antimicrobial concentrations. Scale bar = 20 μm.

Comparative SEM imaging of the EVPL bronchiole tissue infected with PaRP isolate #1 was employed (Figure 5), with concurrent growth of biofilm cells (compared to TVC data) under control conditions (ASM only). In the presence of both concentrations of manuka honey cells were not observed on the surface of bronchioles. Ceftazidime was selected as a representative antibiotic for testing here due to the different effects observed against the selected isolates. In the presence of ceftazidime, bacterial cells were present, however, the addition of varying manuka honey concentrations resulted in the loss of cells from the tissues imaged. It should also be noted that in the presence of 64% (w/v) manuka honey, differences in tissue structure were observed.

## Discussion

Pulmonary *P. aeruginosa* infections are difficult to treat, due to its various AMR and AMT capabilities and the constraints surrounding the effective delivery of antimicrobial agents (Lee and Yoon, 2017). It is therefore crucial that novel antimicrobial agents are identified, especially those with anti-biofilm activity. This study demonstrates that manuka honey was effective at inhibiting a wide range of *P. aeruginosa* isolates (22) associated with CF infection; building on the findings of a previous study (Jenkins et al., 2015). For the first time, it was established that manuka honey reduced the viability of various *P. aeruginosa* isolates in an *ex vivo* infection model (where clinically relevant antibiotics could not), allowing a realistic assessment of the ability of manuka honey to inhibit biofilms in more realistic tissue types. Previous studies (Maddocks et al., 2012; Lu et al., 2014; Liu et al., 2018) have shown antimicrobial efficacy of manuka honey against biofilms grown using conventional *in vitro* methods, however, biofilms grown using *ex vivo* models and ASM are thought to be more representative of clinical biofilms, unlike those formed using conventional *in vitro* methods and microbiological media (Roberts et al., 2015).

Minimum inhibitory concentration testing was completed using methods that are typical in a clinical setting, and whilst it is believed that these methods (which reply on the use of planktonic cells) do not convey what is seen during chronic infection (biofilms) (Roberts et al., 2015), they represent gold standard practice. Whilst methods looking into the Minimum Biofilm Inhibition Concentration (MBIC) could have been used, they are not done routinely in clinic and would not provide any additional information as the MIC and MBIC of manuka honey have been previously observed to be similar (Liu et al., 2015). Conventional MIC values were higher than those seen previously, and is thought to be due to a change in the methodologies, whereby doubling dilutions were used (so as to be in line with EUCAST methodologies) instead of percentage increments (Jenkins et al., 2015). Combining manuka honey with antibiotics yielded varied results based on the sub-inhibitory concentration of manuka honey used, the combined antibiotic, the strain of *P. aeruginosa* and the method used (conventional methods vs. the use of the *ex vivo* model). For the majority of PaRP isolates tested via conventional methods it appeared that manuka honey was non-interactive with the three antibiotics tested, however, some strain specific antagonism and synergism was observed, a stark contrast to previous studies where synergism was widespread (Mandal and Mandal, 2011; Jenkins and Cooper, 2012; Jenkins et al., 2015). The absence of antagonism in the majority of isolates suggests manuka honey could be considered for use alongside conventional antibiotic treatment regimens. In the *ex vivo* models, combinations of manuka honey and clinically relevant antibiotics had anti-biofilm efficacy, reducing viability for the majority of isolates tested, with total inhibition observed in many instances. This puts manuka honey in a strong position as a potential novel therapeutic for chronic upper airway infections, especially where AMR is observed, however, there are some limitations to the *ex vivo* models. Despite similar nutrient cues being used, nutrient availability may differ due to the lack of thick mucus. The physical surface structures of the tissue may differ slightly, especially if it were to degrade during infection which might not be observed during *in vivo* infection of a cystic fibrosis lung.

When considering the use of manuka honey for the treatment of pulmonary infections, ease of application, subsequent reapplications and potential diluting effects are difficult to predict. During interaction studies, multiple sub-inhibitory concentrations of manuka honey were used, allowing dilution effects to be identified. Not only does this allow the efficacy of the manuka honey to be determined at sub-inhibitory concentrations when combined with antibiotics, but identifies any potential problems arising from these combinations. It is critical to take such observations into consideration before progressing into a clinical setting as many of these patients will be undergoing extensive antimicrobial therapy. The specific instances of antagonism at low sub-inhibitory concentrations, is most likely due to the sugars acting as a carbon source, therefore inhibitory concentrations must be maintained. The use of doubling dilutions might have exacerbated this, with previous studies (Roberts et al., 2012; Jenkins et al., 2015) opting for minor percentage reductions in concentrations which does not necessarily convey the range of dilutions that may be encountered *in vivo*. As the reference panel used within this study covers the wide strain diversity seen for *P. aeruginosa* from those that are highly transmissible to those that are extremely virulent, effects against individual strains should also be considered in addition to the overarching effects on the species as a whole. For instance, the synergism of some manuka honey and antibiotic combinations against specific antimicrobial resistant strains (e.g., ciprofloxacin and tobramycin against PaRP isolate #41) suggests combinational therapy could have potential therapeutic benefit, albeit under stringent conditions and may be of benefit where other treatment options fail.

In light of increasing AMR, repurposing antimicrobial agents may provide new treatment options for difficult to treat infections. In this study we have demonstrated the antibacterial and anti-biofilm effects of manuka honey (above the effect seen with antibiotics when used alone) against *P. aeruginosa* grown in two *ex vivo* models, suggesting potential for manuka honey to be re-purposed from its original use as a wound dressing. During routine clinical practice, biofilm-based methods for antimicrobial testing are not used, primarily due to a lack of relevance when it comes to patient prognosis (Coenye et al., 2018), however, they are useful in academic research for the identification of anti-biofilm effects, especially when they better replicate chronic infections and their associated challenges (Waters and Ratjen, 2017). Whilst the anti-bacterial efficacy of manuka honey in an *ex vivo* biofilm model simulating chronic lung infections has been demonstrated, an obvious limitation when it comes to clinical applicability of manuka honey for the treatment of pulmonary infection is delivery. Firstly, Nebulisation of antimicrobials can be both difficult and inefficient, with lower final concentrations (13–64% of the original dose) in the lung environment (Bassetti et al., 2016), therefore manuka honey is unlikely to reach the lung at 64% (w/v) in its current formulation. Concentrations in the region of 40% (w/v) could be achievable; however, efficacy at this concentration needs to be experimentally tested. This potentially brings its own challenges as manuka honey can exert *in vitro* toxicity on human cells (Yabes et al., 2017). The differences between EVPL bronchiole tissue in the presence of 64% (w/v) manuka honey may be subject to these similar “*in vitro”* effects, however, the negative effects observed *in vitro* do not necessarily occur *in vivo* whereby 100% manuka honey is used on open wounds with positive effects, increasing wound healing (Mohamed et al., 2015). Alternatively higher concentration of manuka honey could be readily incorporated into a sinus rinse solution and be used to clear sino-nasal cavity infection reservoirs, preventing the migration of bacteria from the upper airway into the lung (Siegel and Weiser, 2015), and affording terminal CF patients the opportunity to undergo lifesaving lung transplantations.

## Author Contributions

AR and RJ conceived the design of the study, analyzed the data, and wrote the manuscript. AR, LP, and MP carried out the experimental work. LP, MP, DT, and RJ contributed to the final preparation of the manuscript.

## Conflict of Interest Statement

The authors declare that the research was conducted in the absence of any commercial or financial relationships that could be construed as a potential conflict of interest.
