# Supplementary material for: Anti-pseudomonad Activity of Manuka Honey and Antibiotics in a Specialized ex vivo Model Simulating Cystic Fibrosis Lung Infection
Source: Front Microbiol. 2019 Apr 24;10:869. doi: 10.3389/fmicb.2019.00869 (PMC6491927; doi:10.3389/fmicb.2019.00869)
Supplement: Supplementary file 1 [file Data_Sheet_1.PDF]

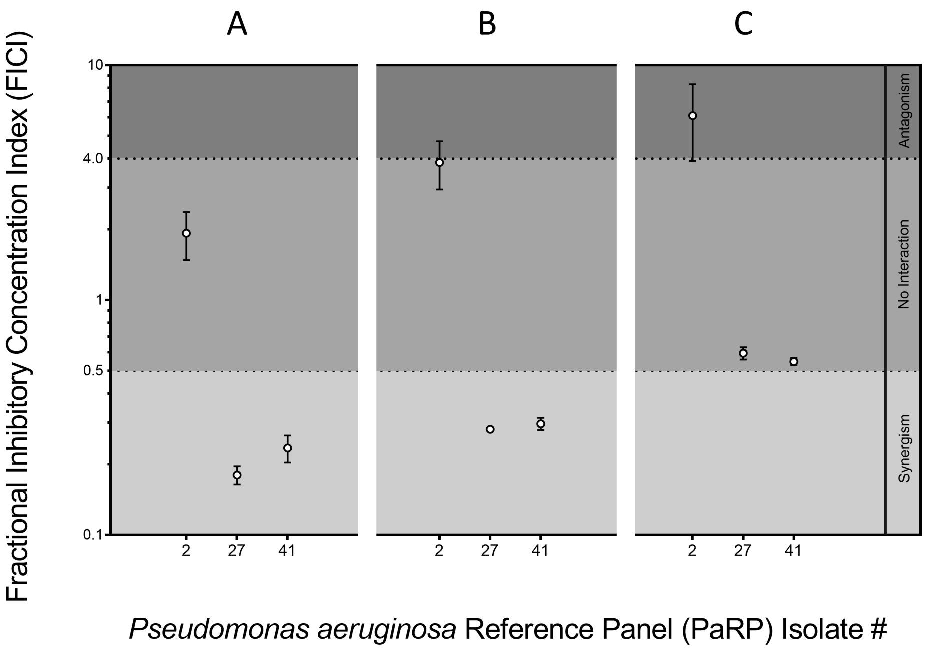

Supplementary Figure 1. Fractional Inhibitory Concentration Index (FICI) of ciprofloxacin, supplemented with 0.125 (**A**), 0.25 (**B**), and 0.5 (**C**) of the manuka honey MIC concentration. The interaction between manuka honey and antibiotics were interpreted as 'synergistic (FICI < 0.5), 'antagonistic' (FICI > 4.0), and 'no interaction' (FICI > 0.5-4.0).

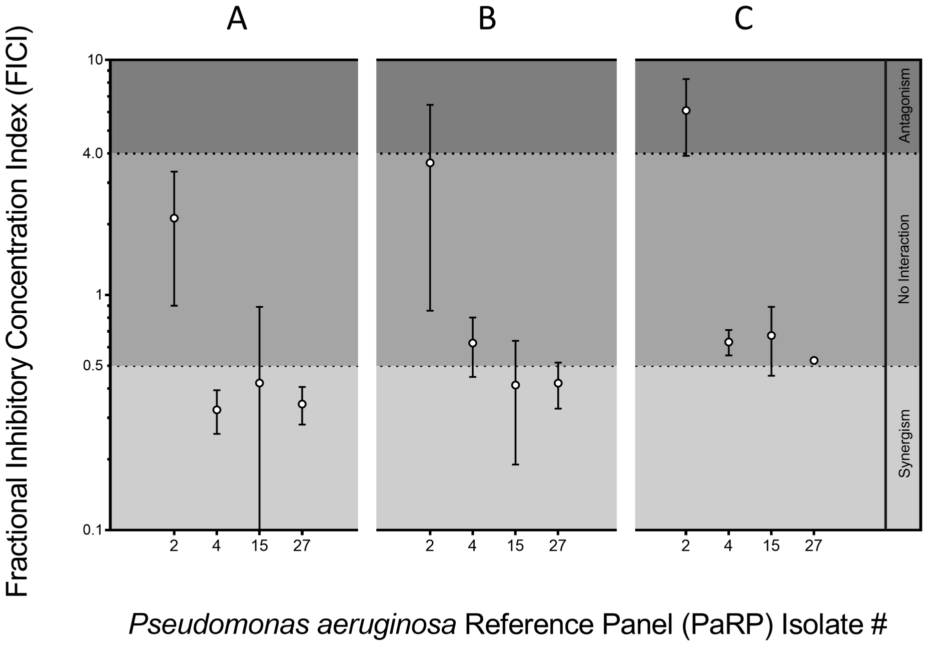

Supplementary Figure 2. Fractional Inhibitory Concentration Index (FICI) of ceftazidime, supplemented with 0.125 (**A**), 0.25 (**B**), and 0.5 (**C**) of the manuka honey MIC concentration. The interaction between manuka honey and antibiotics were interpreted as 'synergistic (FICI < 0.5), 'antagonistic' (FICI > 4.0), and 'no interaction' (FICI > 0.5-4.0).

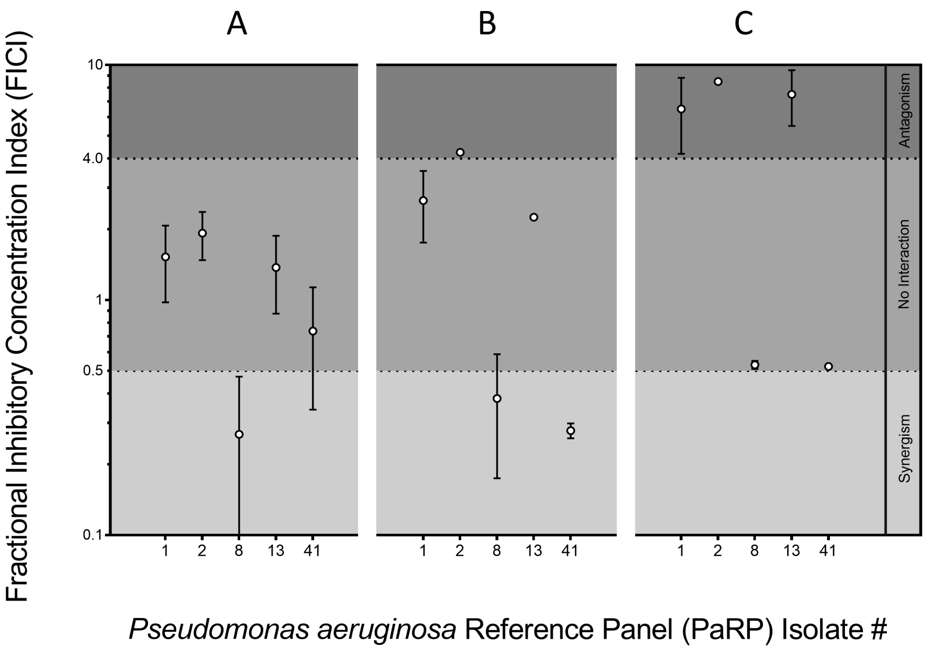

Supplementary Figure 3. Fractional Inhibitory Concentration Index (FICI) of tobramycin, supplemented with 0.125 (**A**), 0.25 (**B**), and 0.5 (**C**) of the manuka honey MIC concentration. The interaction between manuka honey and antibiotics were interpreted as 'synergistic (FICI < 0.5), 'antagonistic' (FICI > 4.0), and 'no interaction' (FICI > 0.5-4.0).

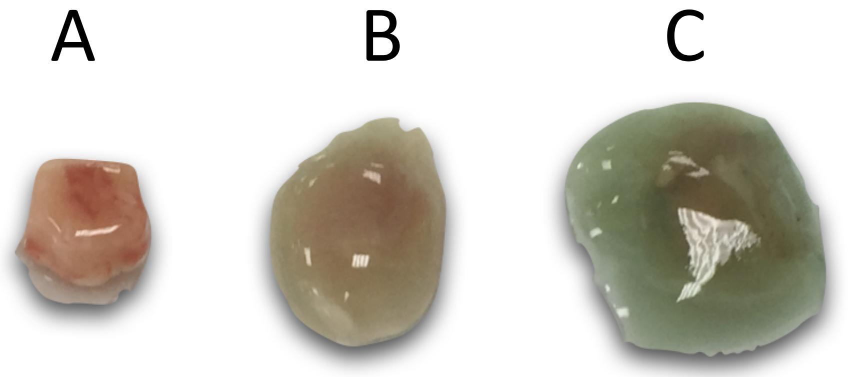

Supplementary Figure 4. Exopolysaccahride production by two *Pseudomonas aeruginosa* isolates grown within $5mm^2$ sections of *Ex vivo* Porcine Lung (EVPL) tissue - uninoculated tissue sample showing control $5mm^2$ tissue cubes (A) comapred to the commonly used reference strain PA01 (B) and the transmissible strain LES B58 (C) which have engulfed the $5mm^2$ tissue cubes.
